# Use of latent profile analysis and k-means clustering to identify student anxiety profiles

**DOI:** 10.1186/s12888-021-03648-7

**Published:** 2022-01-05

**Authors:** Fang Liu, Dan Yang, Yueguang Liu, Qin Zhang, Shiyu Chen, Wanxia Li, Jidong Ren, Xiaobin Tian, Xin Wang

**Affiliations:** 1 School of Public Health, China Medical University, No.77 Puhe Road, Shenyang North New District, Shenyang, 110122 Liaoning China; 2 Nanchong Physical and Mental Hospital (Nanchong Sixth People’s Hospital), No.99 Jincheng Street, Yingshan County, Nanchong, 637000 Sichuan China; 3 Department of Preventive Medicine, North Sichuan Medical College, No.234 Fujiang Road, Nanchong, 637000 Sichuan China; 4 School of Health Management, No.77 Puhe Road, Shenyang North New District, Shenyang, 110122 Liaoning China

**Keywords:** Mental health test, Anxiety, Latent profile analysis, K-means clustering, Chinese

## Abstract

**Background:**

Anxiety disorders are often the first presentation of psychopathology in youth and are considered the most common psychiatric disorders in children and adolescents. This study aimed to identify distinct student anxiety profiles to develop targeted interventions.

**Methods:**

A cross-sectional study was conducted with 9738 students in Yingshan County. Background characteristics were collected and Mental Health Test (MHT) were completed. Latent profile analysis (LPA) was applied to define student anxiety profiles, and then the analysis was repeated using k-means clustering.

**Results:**

LPA yielded 3 profiles: the low-risk, mild-risk and high-risk groups, which comprised 29.5, 38.1 and 32.4% of the sample, respectively. Repeating the analysis using k-means clustering resulted in similar groupings. Most students in a particular k-means cluster were primarily in a single LPA-derived student profile. The multinomial ordinal logistic regression results showed that the high-risk group was more likely to be female, junior, and introverted, to live in a town, to have lower or average academic performance, to have heavy or average academic pressure, and to be in schools that have never or occasionally have organized mental health education activities.

**Conclusions:**

The findings suggest that students with anxiety symptoms may be categorized into distinct profiles that are amenable to varying strategies for coordinated interventions.

**Supplementary Information:**

The online version contains supplementary material available at 10.1186/s12888-021-03648-7.

## Introduction

Anxiety is an emotion characterized by feelings of tension, worried thoughts and physical changes. People with anxiety disorders usually have recurring intrusive thoughts or concerns and may have physical symptoms such as sweating, trembling, dizziness or a rapid heartbeat. Anxiety disorders are often the first presentation of psychopathology in youth and are considered the most common psychiatric disorders in children and adolescents [1–4]. Globally, anxiety is the ninth leading cause of illness and disability among adolescents aged 15–19 years and sixth among those aged 10–14 years [5].

Adolescence is one of the most rapid phases of human development and is associated with marked physical, neurodevelopmental, psychological and social changes [6]. As a unique and formative time period, individuals in adolescence, especially junior and senior high school students, are not only in the pubertal stage but also under significant educational pressure, which places them at a higher risk of suffering from anxiety symptoms. The most prevalent subtypes of anxiety, as previous studies reported, were learning anxiety, body anxiety, self-blaming tendency, phobia anxiety, and sensitivity tendency among primary and secondary students [7, 8].

Promoting psychological well-being and targeting interventions to protect students from adverse experiences and risk factors that may impact their potential to thrive are critical for their physical and mental health. Ideally, mental health promotion and prevention interventions would be tailored to the differing needs of distinct student subgroups rather than treating all students as a homogeneous group [7, 9]. There is a substantial body of evidence regarding subgroup identification in mental health [10–12]. However, these studies seldom focus on adolescents. In addition, most of the studies used only one measure to identify subgroups. Given the absence of a gold standard for statistically validating data clustering results [13], the validation, applicability and stability of such identification results have remained unclear.

Latent profile analysis (LPA) and k-means clustering have commonly been used as grouping methods in previous studies. LPA is a Gaussian finite mixture modelling method used to identify distinct clusters based on participants’ responses to a set of measures or variables using maximum likelihood estimation [14]. K-means clustering is a nonmodel-based method that is not grounded on an underlying statistical model and typically corresponds to discrete optimization algorithms to optimize across a diverse range of objective criteria [13]. Given the markedly different principles underlying these two grouping methods, their results could be mutually verified. Consistent results may provide an opportunity to confirm the accuracy and stability of the results. Grant RW et al. defined distinct patient clinical profiles among the most medically complex patients through latent class analysis, and repeating the analysis using k-means clustering resulted in qualitatively similar groupings. The findings suggested that highly medically complex patient populations may be categorized into distinct patient profiles that are amenable to varying strategies for resource allocation and coordinated care interventions [13]. Hence, the current exploratory study aimed to define distinct student anxiety profiles through LPA and to verify its stability by the k-means clustering method.

## Methods

### Study settings

**Fig. 1:**
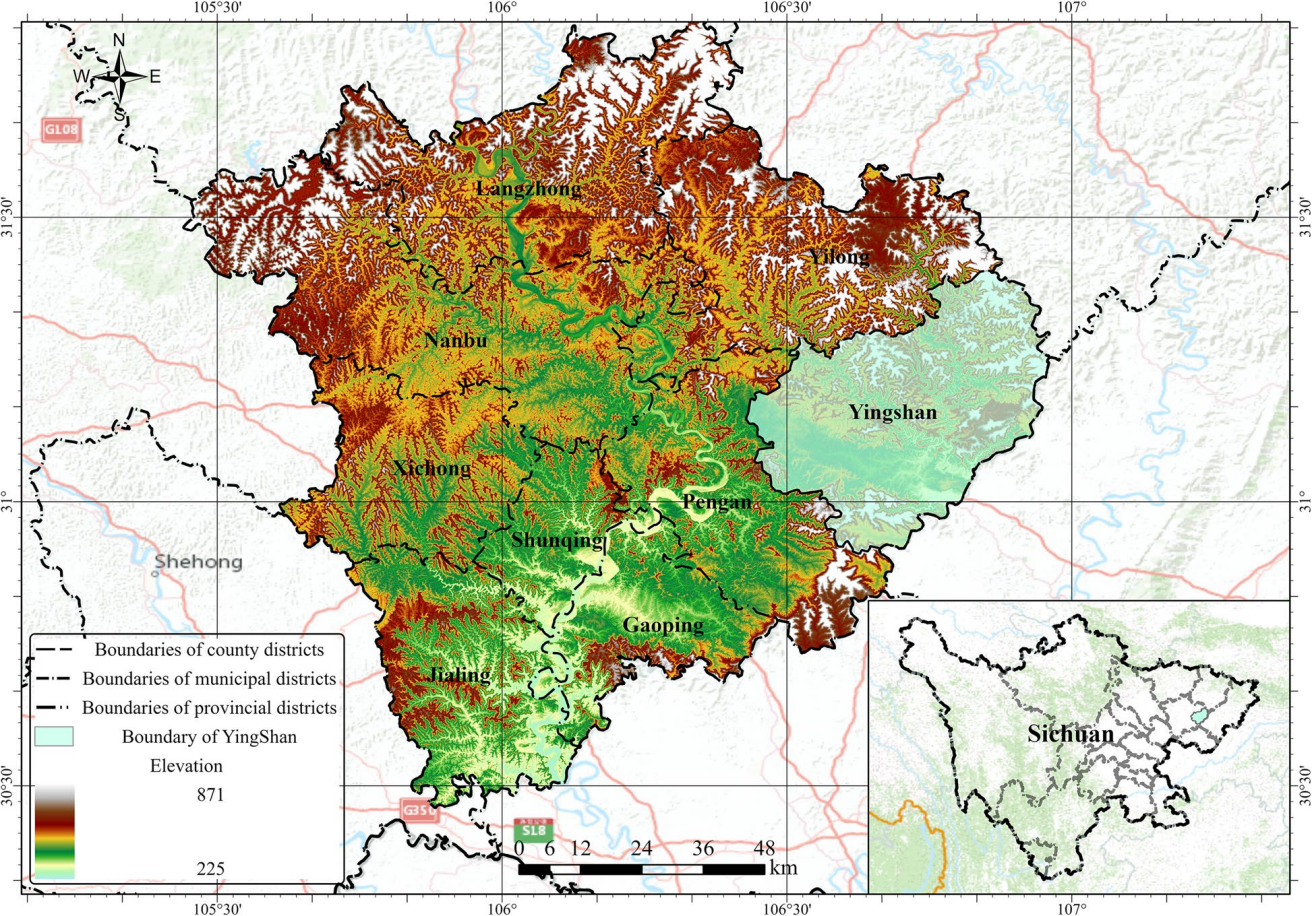
Geographic location and topography map of the sampling areas

This study was conducted in Yingshan County, located northeast of Nanchong City, Sichuan Province, and mainly consists of mountainous terrain. This is a relatively poor and rural region of western China and was considered a poverty-stricken county sequence until 2019. Figure 1 shows the geographic location and topography of the sampling areas.

### Procedures and participants

First, we collected a list of all junior and senior high schools in Yingshan County from the local Bureaus of Education. There were a total of 65 junior and senior high schools, and all were surveyed in our study. Each school had 3 grades (grade 1 to grade 3). Then, we selected two classes at random for each grade level at each school to recruit students. Thus, 390 classes were selected to complete the survey. The inclusion and exclusion criteria for the participants are described below.

The inclusion criteria were as follows: (1) had student status in the school, (2) had sufficient visual and auditory discrimination to complete the psychological tests, and (3) had the ability to give informed consent. The exclusion criteria were as follows: (1) had dropped out of school, and (2) did not agree to participate in the survey.

Finally, a total of 10,525 junior and senior high school students completed the survey. Eliminating 787 students who failed the Mental Health Test (MHT) reliability check from the analysis resulted in a total of 9738 valid responses.

### Data collection

The survey was organized by the Bureau of Education in Yingshan County from July to August 2020. Before the formal investigation, the Bureau organized the principals of the target schools to hold a symposium to introduce the background, aims, process, precautions and quality requirements of this study. Then, the principals sent the relevant content and questionnaires to headteachers in the form of documents. Next, the headteachers held class meetings to explain the study to the students and legal guardians. All participants and legal guardians gave informed consent to participate, and they had the right to refuse and terminate the survey at any time. During the survey, the students were instructed to sit apart and to refrain from discussing any of the questions with other students. Then, an anonymous structured questionnaire was administered to the students in the absence of teachers in the classroom. This study was approved by the ethics committee of Nanchong Physical and Mental Hospital (202002).

### Measures

#### Background characteristics

The individual characteristics included sociodemographic variables (age, gender, grade, character traits, and mode of travel to school), basic family factors (native residential area, family financial conditions, and whether parents work outside), and school-related factors (academic performance, academic pressure and whether the school organized mental health education activities). The questionnaire is provided as an additional file (Additional file 1).

#### Mental health test (MHT)

Anxiety was assessed using the MHT [15] in this study, the most extensively used scale to measure mental health in China [16, 17]. Of the 100 test questions, 10 are reliability questions to detect whether answers were honestly answered. The test was considered invalid if the student answered yes to more than 7 of these questions and was excluded from the analysis. The remaining 90 points can be broken down into eight subcategories [18], each of which represents a specific aspect of anxiety: learning anxiety, personal anxiety, loneliness anxiety, self-blaming tendency, sensitivity tendency, body anxiety, phobia anxiety and impulsive tendency. The participants were asked to indicate whether each of these symptoms described their own condition (0 = no, 1 = yes). A score of greater than 8 on any subcategory was considered clinically high [8]. A total score of 65 or higher indicated a high risk for mental health problems [15]. The scale showed good internal reliability in the present study (Cronbach’s α = 0.951).

### Data analysis

First, basic descriptive statistics to describe the participants’ demographic characteristics were employed.

Second, we used LPA to assign students to their most likely group based on their anxiety profile. We assumed that each student belonged to one of a set of *n* latent profiles, the number or size of which were unknown a priori [19]. Several fit indicators were used to assess goodness-of-fit and to determine the optimal number of latent profiles. The Akaike information criterion (AIC), Bayesian information criterion (BIC) and adjusted Bayesian information criterion (aBIC) were mainly indicators used to evaluate the quality of different models, and lower values indicated better model fit [20]. Entropy is a measure of classification accuracy, and higher values indicated better classification quality. The Lo-Mendell-Rubin (LMRT) and bootstrap likelihood ratio test (BLRT) are tests of significance between two models with *k* classes against *k*-1 classes; a significant *p* valu*e* indicated that the *k* class was better [11]. Moreover, it should be noted that the interpretability and practical implications for practitioners were also considered in the determination of the final model [21]. For each student, the posterior probability of belonging to each of the profiles was calculated, and students were assigned to the latent profile with the largest probability. After determining the best latent profile solution, we compared the eight anxiety subcategories among the latent profile groups by using analysis of covariance (ANOVA).

Third, k-means clustering was used to identify the anxiety profiles of students who would also be classified most frequently in the same cluster. The optimal cluster number solution was determined by the elbow test. The elbow method ran k-means clustering on the dataset for a range of values for *k*, and, for each value of *k,* the sum of squared errors (SSE) was calculated. The optimal number is the elbow position [22]. Next, the Sankey diagram was used to visualize the degree of overlap between the LPA results and k-means clustering results.

Finally, the latent profile subtypes were compared using chi-square tests. Multinomial logistic regression was used to determine which factors predicted different latent profiles of anxiety students. Additionally, LPA was performed by Mplus 7.4, and other statistical analyses were conducted by using RStudio. A two-sided *P* value < 0.05 was considered statistically significant.

## Results

### Descriptive statistics

The mean age was 14.46 ± 1.61 years (range: 8–20). Of all participates (*n* = 9738), 51.1% were females. The great majority were junior students (74.7%), and 41.3% were introverted. In addition, 32.6% were in-residence, and 41.5% came from rural areas. Only 3.8% reported good family financial status. In addition, 41.4% of students’ parents work outside. Approximately one-third reported lower academic performance (36.7%) and perceived heavy academic pressure (33.3%). Only 35.2% of the schools often organized mental health education activities. A total of 11.9% of the participants scored above the cut-off for mental problems (MHT ≥ 65). These data are summarized in Table 1.

**Table 1:** Demographics of survey participants (*N* = 9738)

Variable	Total	Percentage (%)
**No. (%)**	9738	100
***Gender***		
Female	4973	51.1
Male	4765	48.9
***Grade***		
Senior	2468	25.3
Junior	7270	74.7
**Character traits**		
Introversion	4019	41.3
Extroversion	5719	58.7
***Mode of travel to school***		
Nonresident	6567	67.4
In-residence	3171	32.6
***Residence***		
Urban area	3989	41.0
Town	1705	17.5
Rural area	4044	41.5
***Family financial conditions***		
Good	367	3.8
Average	7287	74.8
Poor	2084	21.4
***Whether parents work outside***		
Both outside	4030	41.4
Father or mother outside	2747	28.2
Both at home	2961	30.4
***Academic performance***		
Upper	1145	11.8
Medium	5023	51.6
Lower	3570	36.7
***Perceived academic pressure***		
Light	1110	11.4
Average	5384	55.3
Heavy	3244	33.3
***Has the school organized mental health education activities***		
Often	3423	35.2
Occasionally	5007	51.4
Never	1308	13.4
***Mental Health Test***		
0–55	7392	75.9
56–64	1183	12.2
65–90	1163	11.9

### Latent profile analysis

Models with one through five profiles (k = 1–5) were compared to identify the optimal number of profiles (Table 2). The results showed that the AIC, BIC and aBIC decreased with an increasing number of classification profiles. The 1-class and 2-class models had the largest AIC, BIC and aBIC values, suggesting that these models fit the data worse than the other models. Regarding the 3-class and 4-class models, the entropy value of the 3-class model was higher, indicating that the 3-class model fit the data better than the 4-class model did. Then, regarding the comparison between the 3-class and 5-class models, the 5-class model had lower AIC, BIC and aBIC values and higher entropy than the 3-class model and indicating that the 5-class model was better. However, after comprehensive consideration, we selected the 3-class model because it was concise and had better interpretability than the 5-class model. From a practical standpoint, it is impractical and not easy to divide students into five subgroups for development of targeted measures. This also showed that LPA involves, to a certain extent, a great deal of subjectivity, which requires other ways to compensate for this defect.

**Table 2:** Fitness indicators of different latent profiles

Models	AIC	BIC	aBIC	Entropy	LMR, ***p*** value	BLRT, ***p*** value	Mixing ratios
1-class	161,370.347	161,485.288	161,434.442	–	–	–	–
2-class	140,064.098	140,243.693	140,164.246	0.851	0.000	0.000	59.191%/40.809%
3-class	130,728.595	130,972.844	130,864.797	0.911	0.000	0.000	38.129%/29.513%/32.358%
4-class	128,706.354	129,015.257	128,878.610	0.848	0.000	0.000	20.384%/32.050%/17.396%/30.170%
5-class	122,801.884	123,175.441	12,301.193	0.945	0.000	0.000	24.502%/30.304%/13.216%/12.569%/19.409%

*Abbreviations*: *AIC* Akaike information criterion, *BIC* Bayesian information criterion, *aBIC* adjusted Bayesian information criterion, *LMR* Lo-Mendell-Rubin, *BLRT* bootstrap likelihood ratio test

**Fig. 2:**
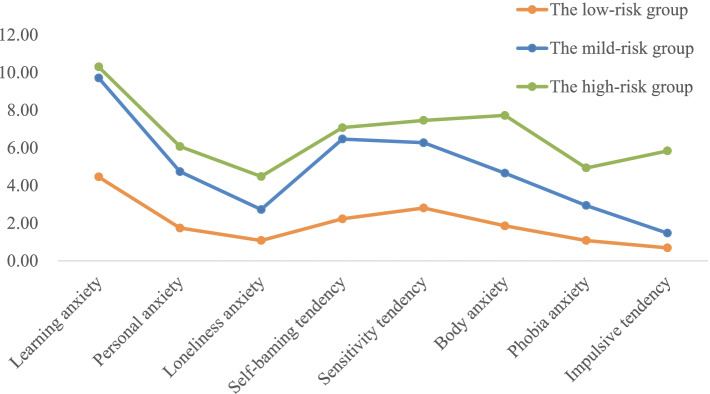
Latent profile plot based on the MHT for students (the x-axis shows indicator variables used for the LPA, while the y-axis represents the mean score for the eight subcategories. The three lines show symptom patterns for the three anxiety subcategories)

As shown in Fig. 2, grouping the students based on anxiety yielded 3 profiles: the low-risk group, the mild-risk group and the high-risk group, which comprised 29.5, 38.1 and 32.4% of the sample, respectively. No crossing was observed among the three lines, suggesting that the modelled subtypes differed in symptom profiles. Notably, these three curves showed similar trends. Each group of participants had high learning anxiety. In addition, personal anxiety, self-blaming tendency, sensitivity tendency and body anxiety were relatively high, while loneliness anxiety and phobia anxiety were low.

ANOVA was conducted validate the anxiety latent profiles (Table 3). The three latent profiles revealed different patterns of correlations with the eight anxiety subcategories. In particularly, the high-risk group demonstrated higher mean scores than the other two latent profiles. The anxiety scores for the eight dimensions in the low-risk group were significantly lower than those in the mild-risk group and the high-risk group, while the anxiety scores for the eight dimensions in the mild-risk group were significantly higher than those in the low-risk group and significantly lower than those in the high-risk group.

**Table 3:** Mean comparisons across the three latent profiles

	low-risk group	mild-risk group	high-risk group	F	*p* value
	Mean ± SD	Mean ± SD	Mean ± SD		
Learning anxiety	4.46 ± 2.855	9.72 ± 2.696	10.30 ± 3.069	3800.350	0.000
Personal anxiety	1.75 ± 1.621	4.74 ± 2.018	6.08 ± 2.219	3746.500	0.000
Loneliness anxiety	1.09 ± 1.361	2.73 ± 2.240	4.49 ± 2.682	1819.440	0.000
Self-blaming tendency	2.23 ± 2.164	6.47 ± 2.314	7.08 ± 2.392	3977.063	0.000
Sensitivity tendency	2.81 ± 2.117	6.27 ± 1.824	7.46 ± 1.744	4893.964	0.000
Body anxiety	1.86 ± 1.740	4.66 ± 2.314	7.72 ± 2.887	4598.731	0.000
Phobia anxiety	1.08 ± 1.523	2.94 ± 2.417	4.94 ± 2.742	2096.410	0.000
Impulsive tendency	0.70 ± 1.016	1.48 ± 1.117	5.84 ± 1.599	14,985.950	0.000
Total	19.65 ± 9.808	43.90 ± 9.382	59.12 ± 12.406	10,609.958	0.000

### K-means clustering results

**Fig. 3:**
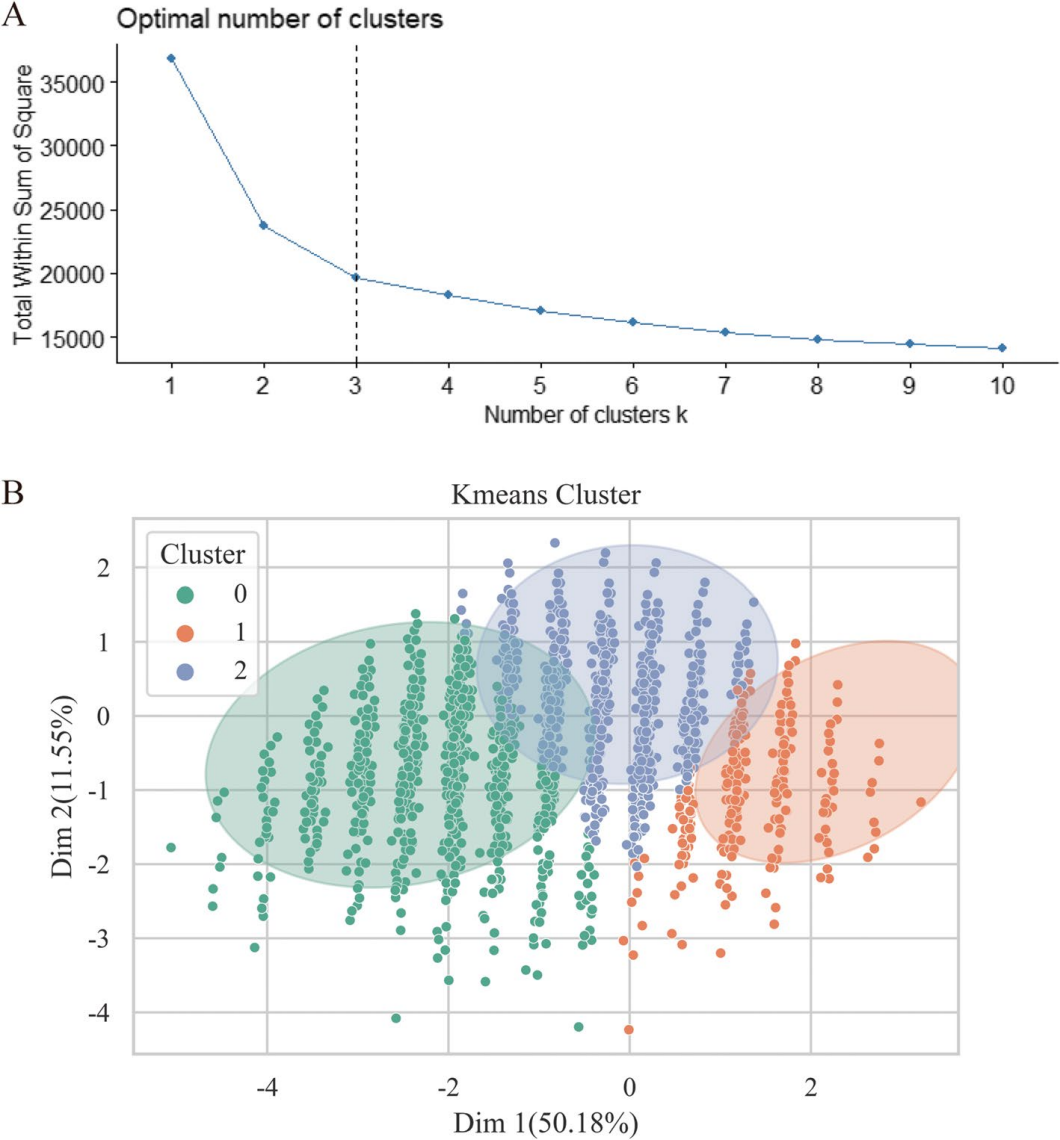
Elbow test for k-means analysis and k-means clustering results (A: visual inspection of the plot demonstrates that the optimal number of clusters is 3 in the elbow test. B: The k-means clustering results show a high degree of similarity with regard to clustering designations. As the clusters were generated on 8 axes (one for each anxiety subcategory), these axes were condensed into two distilled axes (dimensionality reduction), represented here by “Dim1” and “Dim 2” to represent the greatest concordance between the data)

The elbow test depicted in Fig. 3(A) showed that the greatest decrease in slope for SSE across sequential clusters was from *k* 2–3 to *k* 3–4. Therefore, we selected *k* = 3 as the optimal number of categories suited for our data and then used k-means clustering to cluster the data into 3 clusters. As shown in Fig. 3(B), Cluster 1, including 2989 (30.7%) of the 9738 clustered students, was characterized by relatively low anxiety. Cluster 2, including 4109 (42.2%) students, was characterized by mild anxiety. The 2640 (27.1%) students in Cluster 0 showed higher anxiety than the students in other clusters.

### Comparison of LPA results with k-means clustering results

**Fig. 4:**
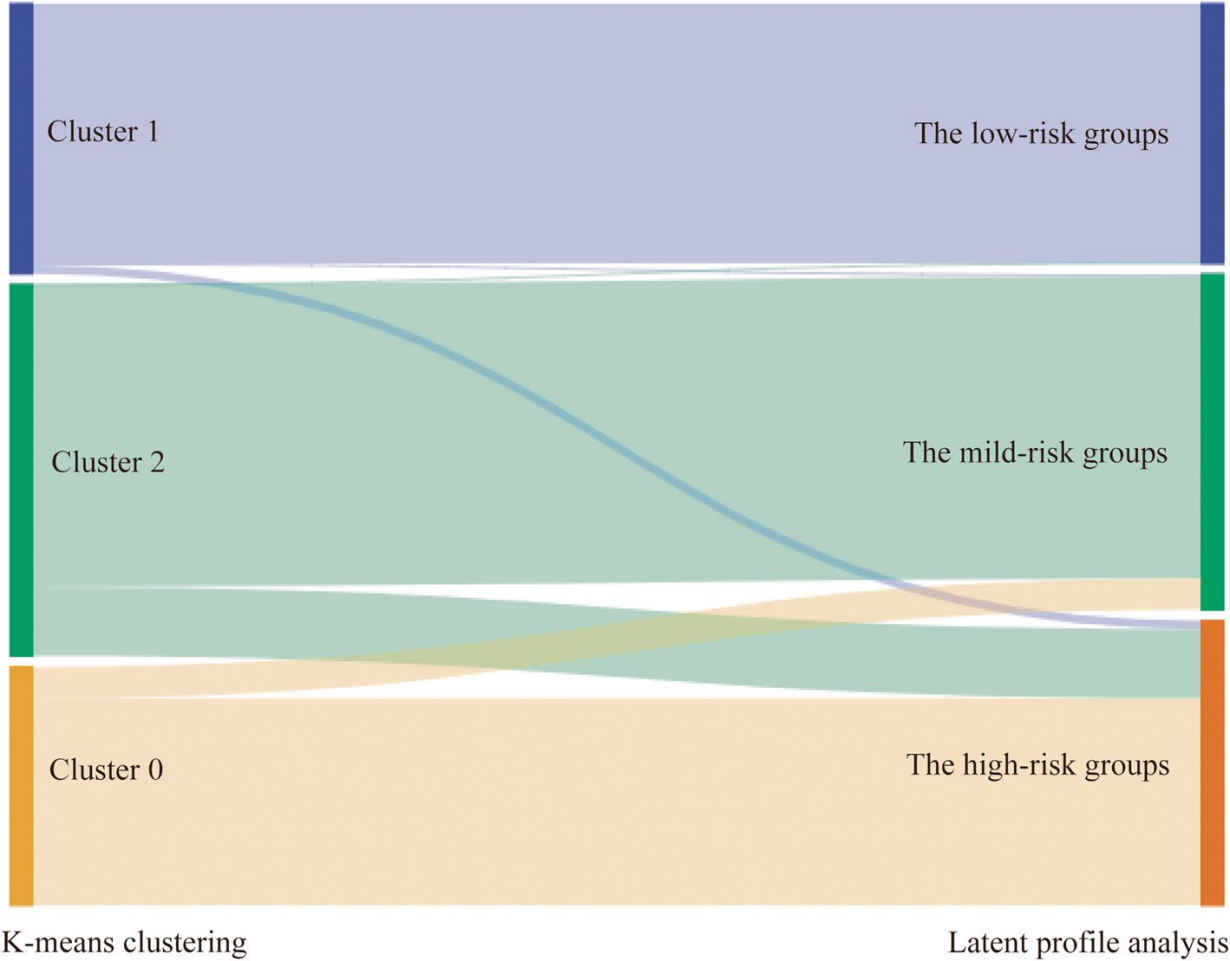
A Sankey diagram illustrating the overlap between the LPA results and k-means clustering results (rectangles denote states in the two classifications; coloured banners denote flows from one state to another across the two classifications; the widths of the banners are proportional to the overlapping number of students with a particular anxiety profile in each segment)

We investigated the extent to which patients assigned to these 3 clusters matched the 3 profiles derived from the LPA. The width of the flow line represents the degree of overlap. The more overlap between the two results, the more reliable the classification. As shown in Fig. 4, most students in a particular k-means cluster were primarily in a single LPA-derived student profile. For example, 96.12% of the students in the Cluster 1 overlapped with those in the low-risk LPA group, and 81.33% of the students in Cluster 2 were in the mild-risk LPA group. In addition, 86.86% of the students in Cluster 0 were in the high-risk LPA group. There was substantial overlap in the LPA results and k-means clustering results, which verified, to a certain degree, that it was reasonable to yield 3 profiles.

### Multinomial logistic regression analysis of latent profiles of anxiety students

**Fig. 5:**
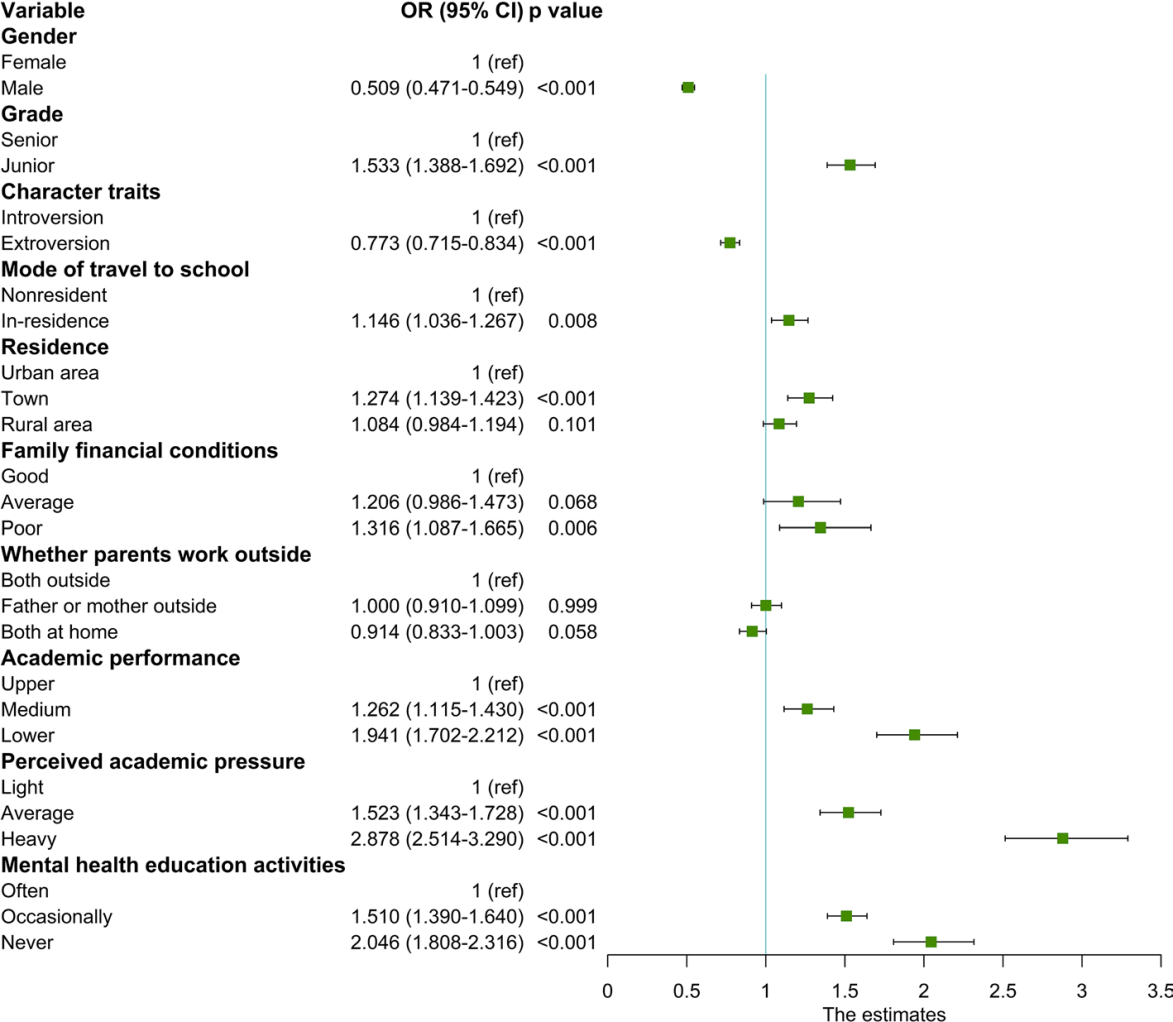
Multinomial ordinal logistic regression analysis of different latent profiles

Based on the latent profile analysis, we analysed relevant factors associated with these profiles. As shown in univariate analyses, sociodemographic factors, basic family factors and school-related factors were all significantly different across the three groups (*P* < 0.05) (Additional File 2). The significant factors in the univariate analyses were used as independent variables for multinomial ordinal logistic regression analysis (Fig. 5). In the multivariate models, males (OR = 0.509, 95% CI = 0.471–0.549) and extroverted students (OR = 0.773, 95% CI = 0.715–0.834) reported lower anxiety. Students who were at the junior school (OR = 1.533, 95% CI = 1.388–1.692), in-residence (OR = 1.146, 95% CI = 1.036–1.267), living in a town (OR = 1.274, 95% CI = 1.139–1.423), and had poor family financial conditions (OR = 1.316, 95% CI = 1.087–1.665) reported higher anxiety. Regarding academic performance, lower or medium academic performance (OR = 1.941, 95% CI = 1.702–2.212; OR = 1.262, 95% CI = 1.115–1.430, respectively) increased the risk of anxiety compared with higher academic performance. Regarding academic pressure, heavy or average academic pressure (OR = 2.878, 95% CI = 2.514–3.290; OR = 1.523, 95% CI = 1.343–1.728, respectively) increased the risk of anxiety compared with light academic pressure. The students in schools that never or occasionally organized mental health education activities had an increased risk of anxiety (OR = 2.046, 95% CI = 1.808–2.316; OR = 1.510, 95% CI = 1.390–1.640, respectively). Whether parents worked outside was not associated with either group.

## Discussion

In this study, we investigated 9738 junior and senior high school students who completed the MHT. Before the survey, we received strong support from the local Bureau of Education, principals, and teachers, which ensured the orderly development of the survey, strengthened students’ attention to the survey and improved the quality of the investigation. Based on individual responses to different items, we identified 3 anxiety profiles through the application of LPA, namely, the low-risk group, the mild-risk group, and the high-risk group, and verified the stability of these groups by k-means clustering. The students across the three groups scored significantly different on eight anxiety subcategories, with higher scores being observed in the high-risk group. Multinomial ordinal logistic regression analysis was performed to identify influencing factors. Findings from the current study may provide guidance for the formulation of prevention strategies.

### The rationality for grouping

LPA and k-means clustering are commonly used to determine latent subgroups in diverse populations. Guo L et al. identified three latent classes of health behaviour reported by people at high risk of stroke, and this study had significance for the promotion of adaptive health behaviour in individuals at high risk of stroke [23]. Liu et al. categorized medical students based on their depression and anxiety symptoms and suggested that school administrative departments carry out targeted psychological counselling based on different subgroups to promote the physical and mental health of medical students [10]. Warren CM et al. employed LPA to identify whether psychosocial factors clustered together in different patterns among early adolescents and suggested that adolescents engaging in obesogenic and substance use behaviours may share common profiles of psychosocial risk [24]. Ranti D et al. defined novel phenotypes of total hip or total knee arthroplasty patients with k-means clustering to improve upon existing risk stratification among those undergoing joint arthroplasty for preoperative hydration as a targeted intervention to expedite recovery [25]. In the present study, based on the MHT data from junior and senior high school students, we employed LPA to categorize students into three anxiety subgroups and verified the results by k-means clustering. There was substantial overlap (81.33–96.12%) between the two sets of results, implying a certain degree of stability in the classification in our study. Findings from these studies provide guidance for the formulation of targeted interventions for different kinds of groups.

### The practical significance of the grouping results

In the present study, the students in the three subgroups showed a graded difference in scores on each subscale of the MHT, suggesting a marked heterogeneity among the students regarding mental health. The grouping can reflect the actual situation and have actual meaning. This result is very important for the development of effective intervention strategies, especially in areas where prevention and control resources are particularly scarce. Resources can be prioritized for the high-risk group. At the same time, the findings have also increased the relevance of future screening and follow-up work. Most previous intervention measures have been directed toward the whole population [7, 26, 27], and these effects may not be obvious.

A high score on learning anxiety was observed in all three subgroups, even in the low-risk group, which indicated that all students might experience high level of pressure involving education. This was consistent with previous reports [8, 28]. In the context of a competitive Chinese education system, excessive focus on grades might drive high rates of learning anxiety [7]. In China, especially poor and rural regions, people subconsciously believe that only learning can change their destiny, and thus, they show an eager desire and expectation for learning [29]. Additionally, the participants in our study were all junior and senior high school students who were under significant pressure in preparation for senior high school entrance examinations and college entrance examinations [30]. In addition, with the onset of puberty, other psychological changes, such as personal anxiety, self-blaming tendency, sensitivity tendency and body anxiety, cannot be ignored. These forms of anxiety were also at a higher level.

Given the demonstrated heterogeneity among the students, efforts to manage and tailor interventions may be guided by the needs suggested for each of the different profiles. Teachers may pay more attention to the high-risk group and help students actively adjust aspects of their psychology. Additionally, strategies could be developed to address shared needs across groups, as each group of students had high learning anxiety. Taking effective measures to reduce study pressure can greatly improve students’ overall mental health. Study support groups can be set up to help students who have lower academic performance improve their grades and promote mutual help and mutual progress among students. In addition, the main point that should be considered in health programmes is the planning of programmes to strengthen psychological counselling and to improve students’ acceptance of changes in puberty as a physiological process and a positive and correct perception of self-body image [31].

### The application value of the grouping results

In the present study, the results of multinomial ordinal logistic regression showed that the students in the high-risk group tended to be female, in junior high school, introverted, in-residence, and living in the town. They had poor family financial conditions, lower or medium academic performance, and heavy or average academic pressure. Additionally, their schools had never or occasionally organized mental health education activities. These findings were consistent with previous studies [7, 16]. These results also revealed that the latent profiles can accurately identify the types of students. This again showed that this classification is reasonable and has application value. Identifying the characteristics of students in distinct groups is critical for screening. Based on the influencing factors, the risk factors in this population could be noticed by parents, teachers and society. Anxiety screening should be included in students’ physical examination, and teachers should establish student mental health files, evaluate students’ mental health, and pay special attention to students with abnormal results. In addition, mental health education activities should be regularly held to help students better understand themselves and promote self-regulation.

### Limitations

Some study limitations need to be acknowledged. First, the current sample was collected only in one place, which limits generalization to other places. Second, regarding the comparison between k-means clustering and LPA results, we only describe the percentages of coincidence as a measure of the reasonableness of the classification patterns, which may be an easy and a subjective approach. Finally, the students’ mental health problems included not only anxiety but also other psychological problems that were not taken into consideration in our study. This may have led to an underestimation of students’ psychological problems.

## Conclusions

In summary, we used LPA and k-means clustering to identify student anxiety profiles. The findings suggested that students with anxiety symptoms could be categorized into distinct profiles that were amenable to varying strategies for management and targeted interventions. Future work could involve creating algorithms to identify students based on addressable needs, modelling to predict when students might be on a trajectory towards one of these profiles, categorizing the major pathways leading to higher anxiety, and identifying preventive interventions that aim to slow such transitions.

## Supplementary Information

**Additional file 1.** English version and Chinese version of Chinese Adolescent Physical and Mental Health Questionnaire.

**Additional file 2.** Univariate analysis of different latent profiles of anxiety students

## Data Availability

The original data are available upon request to the corresponding author.

## Abbreviations

MHT: Mental Health Test
LPA: Latent Profile Analysis
ANOVA: Analysis of covariance
SSE: The Sum of Squared Errors
AIC: Akaike information criterion
BIC: Bayesian information criterion
aBIC: Adjusted Bayesian information criterion
LMR: Lo-Mendell-Rubin
BLRT: Bootstrap likelihood ratio test
