# Correction: Use of latent profile analysis and k-means clustering to identify student anxiety profiles

**DOI:** 10.1186/s12888-022-04066-z

**Published:** 2022-06-23

**Authors:** Fang Liu, Dan Yang, Yueguang Liu, Qin Zhang, Shiyu Chen, Wanxia Li, Jidong Ren, Xiaobin Tian, Xin Wang

**Affiliations:** 1 School of Public Health, China Medical University, No.77 Puhe Road, Shenyang North New District, Shenyang, 110122 Liaoning China; 2 Nanchong Physical and Mental Hospital (Nanchong Sixth People’s Hospital), No.99 Jincheng Street, Yingshan County, Nanchong, 637000 Sichuan China; 3 Department of Preventive Medicine, North Sichuan Medical College, No.234 Fujiang Road, Nanchong, 637000 Sichuan China; 4 School of Health Management, China Medical University, No.77 Puhe Road, Shenyang North New District, Shenyang, 110122 Liaoning China

**Correction: BMC Psychiatry 22, 12 (2022)**

**https://doi.org/10.1186/s12888-021-03648-7**

Following the publication of the original article [1], the authors identified an error in affiliation 4.

The incorrect affiliation is: 4 School of Health Management, No.77 Puhe Road, Shenyang North New District, Shenyang 110122 Liaoning, China.

The correct affiliation is: 4 School of Health Management, China Medical University, No.77 Puhe Road, Shenyang North New District, Shenyang 110122, Liaoning, China.

The original article [1] has been corrected.
