# Supplementary material for: Use of latent profile analysis and k-means clustering to identify student anxiety profiles
Source: BMC Psychiatry. 2022 Jan 5;22:12. doi: 10.1186/s12888-021-03648-7 (PMC8728926; doi:10.1186/s12888-021-03648-7)
Supplement: Supplementary file 1 — Additional file 1. English version and Chinese version of Chinese Adolescent Physical and Mental Health Questionnaire. [file 12888_2021_3648_MOESM1_ESM.docx]

Additional File 1

**Chinese Adolescent Mental Health Questionnaire**

The purpose of this survey is to understand the mental health status of adolescents. Please tick the corresponding answer according to your actual situation and real experience. Your objective and true answer will be helpful for us to develop measures to promote the healthy growth of adolescents. Thank you for your cooperation. This survey is anonymous, and all information is strictly confidential, please do not have any concerns.

**Part Ⅰ: Sociodemographic Information**

1. Age: _______
2. Gender:

① Female

② Male

3. Grade:

① Senior

② Junior

4. Character traits

① Introversion

② Extroversion

5. Mode of travel to school:

① Nonresident

② In-residence

6. Residence:

① Urban area

② Town

③ Rural area;

7. Family financial conditions:

① Good

② Average

③ Poor

8. Whether parents work outside:

① Both outside

② Father or mother outside

③ Both at home

9. Academic performance:

① Upper

② Medium

③ Lower

10. Perceived academic pressure:

① Light

② Average

③ Heavy

11. Has the school organized mental health education activities

① Often

② Occasionally

③ Never

**Part Ⅱ: Mental Health Test**

This test is to investigate your mood and feelings. It is not a test of intelligence or learning ability. It has nothing to do with academic performance, and there is no good or bad answer.

① Please answer truthfully as you usually think;

② Every question must be answered, but only one answer can be selected; if it is difficult to decide, please choose the closest answer to you;

③ If you don’t understand, you can raise your hand and ask the teacher;

④ There is no limit to the answering time, but don’t think too much about it. Please write down the answer you originally wanted;

⑤ Each question in this test has only two alternative answers: "Yes" and "No".

Tick in the options (√).

| **Mental Health Test** | **Yes** | **No** |
| --- | --- | --- |
| 1. Do you always think about tomorrow lessons when you go to bed at night? |  |  |
| 1. Do you feel uneasy about asking yourself when the teacher asks the whole class questions? |  |  |
| 1. Do you get nervous when you hear about "going to take an exam"? |  |  |
| 1. Do you feel very unhappy when you don't do well in the exam? |  |  |
| 1. Do you always worry when your academic performance is not good? |  |  |
| 1. Do you get nervous during exams when you can't remember what you have learned? |  |  |
| 1. Do you always feel uneasy before you know your grades after the exam? |  |  |
| 1. Do you worry that you will fail in the exam whenever you encounter it? |  |  |
| 1. Do you want every exam to go well? |  |  |
| 1. Do you always worry about not finishing the task before you finish it? |  |  |
| 1. Are you always afraid of making mistakes when you read the text aloud in front of everyone? |  |  |
| 1. Do you think the academic results obtained in school are always unreliable? |  |  |
| 1. Do you think you are more worried about studying than others? |  |  |
| 1. Have you ever had a dream that you failed in the exam? |  |  |
| 1. Have you ever dreamed of being reprimanded by your parents or teachers when your academic performance is not good? |  |  |
| 1. Do you often feel that some classmates speak ill of you behind your back? |  |  |
| 1. Do you always think hard and take it to heart after being criticized by your parents? |  |  |
| 1. Don't you want to do it again if you lose to each other in the game or competition with others? |  |  |
| 1. Do you hate people talking about you behind your back? |  |  |
| 1. Do you blush in front of everyone or when asked questions by teachers? |  |  |
| 1. Are you worried about asking you to do class work? |  |  |
| 1. Do you always feel as if someone is paying attention to you? |  |  |
| 1. Will you be nervous if someone is paying attention to you when you are working or studying? |  |  |
| 1. Are you in a bad mood when you are criticized? |  |  |
| 1. Do you always feel uneasy when you are criticized by your teacher? |  |  |
| 1. Do you laugh less though other students are laughing? |  |  |
| 1. Do you think it is better to play in your own home when you go to your classmate’s home? |  |  |
| 1. Do you feel alone though you are with the crowd? |  |  |
| 1. Do you think it is better to play by yourself alone than to play with your classmates? |  |  |
| 1. Do you not want to join in when other students are talking? |  |  |
| 1. Do you feel that you are redundant when you are with everyone? |  |  |
| 1. Do you hate taking part in sports games and theatrical performances? |  |  |
| 1. Do you have few friends? |  |  |
| 1. Do you dislike talking with others? |  |  |
| 1. Do you feel scared in a crowded place? |  |  |
| 1. Do you always think you are not doing well when you lose in volleyball, basketball or other collective competitions? |  |  |
| 1. Do you always think you are not good after being criticized? |  |  |
| 1. Do you think you have done something wrong when others laugh at you? |  |  |
| 1. Do you always think it's because you don't study hard when you don't do well in school? |  |  |
| 1. Do you always think it's your own responsibility when you fail? |  |  |
| 1. Do you think it is mainly your own fault when everyone is blamed? |  |  |
| 1. Do you pay special attention when you make mistakes when you take part in table tennis, badminton, radio exercises or other sports competitions? |  |  |
| 1. Do you think you can't cope with difficult things? |  |  |
| 1. Do you sometimes regret: "I wish I hadn't done that thing"? |  |  |
| 1. Do you always think it is your fault after quarreling with your classmates? |  |  |
| 1. Do you always want to do something good for your class? |  |  |
| 1. Do your often have thoughts wandering when you study? |  |  |
| 1. Are you worried that others will break it when you lend something to others? |  |  |
| 1. Are you very upset when something goes wrong? |  |  |
| 1. Are you very worried about someone in your family getting sick or dying? |  |  |
| 1. Have you ever seen a dead person in your dreams? |  |  |
| 1. Are you particularly sensitive to radio or car sounds? |  |  |
| 1. Do you always feel as if something is not done well? |  |  |
| 1. Are you always worried that something unexpected will happen? |  |  |
| 1. Do you always hesitate when deciding what to do? |  |  |
| 1. Do you often sweat on your hands? |  |  |
| 1. Do you blush when you are shy? |  |  |
| 1. Do you often have headaches? |  |  |
| 1. Are you always nervous when you are asked questions by your teacher? |  |  |
| 1. Do you often feel heart beat though you don't take part in sports? |  |  |
| 1. Do you get tired easily? |  |  |
| 1. Are you very reluctant to take medicine? |  |  |
| 1. Do you have difficulty falling asleep at night? |  |  |
| 1. Do you always feel as if there is something wrong with your body? |  |  |
| 1. Do you often think that your body and face are uglier than others? |  |  |
| 1. Do you often feel bad about your stomach? |  |  |
| 1. Do you often bite your nails? |  |  |
| 1. Do you often lick your fingers? |  |  |
| 1. Do you often feel difficulty breathing? |  |  |
| 1. Do you go to the bathroom more often than others? |  |  |
| 1. Are you afraid to go to high places? |  |  |
| 1. Are you afraid of many things? |  |  |
| 1. Do you often have nightmares? |  |  |
| 1. Are you timid? |  |  |
| 1. Are you afraid of sleeping alone in your room at night? |  |  |
| 1. Are you scared when you drive through the tunnel or pass high bridge? |  |  |
| 1. Do you like to sleep with the lights on all night? |  |  |
| 1. Were you terrified to hear the thunder? |  |  |
| 1. Are you very afraid of darkness? |  |  |
| 1. Do you often feel someone following you? |  |  |
| 1. Do you often get angry? |  |  |
| 1. Don't you want to get good grades? |  |  |
| 1. Do you often suddenly want to cry? |  |  |
| 1. Have you ever told a lie before? |  |  |
| 1. Do you sometimes feel that it is better to die? |  |  |
| 1. Have you never stood up once? |  |  |
| 1. Do you often want to shout? |  |  |
| 1. Can you keep secret that others don’t want you tell to say? |  |  |
| 1. Do you sometimes think of going far away by yourself? |  |  |
| 1. Are you always polite? |  |  |
| 1. Do you want to take immediate revenge when you have been spoke ill of others? |  |  |
| 1. Do you do everything that your teacher or parents say? |  |  |
| 1. Will you litter or smash things when you are unhappy? |  |  |
| 1. Have you ever been angry? |  |  |
| 1. Do you have to get what you want? |  |  |
| 1. Do you feel particularly happy when the teacher finishes class early for the lessons you don't like? |  |  |
| 1. Do you often want to jump from a high place? |  |  |
| 1. Are you very affectionate to anyone? |  |  |
| 1. Do you often fidget with impatience? |  |  |
| 100. Do you like everyone you don't know? |  |  |

**中学生心理健康调查问卷**

**本调查的目的是了解青少年的心理健康状况。请根据您的实际情况勾选相应的答案。您客观真实的回答将对我们制定促进青少年健康成长的措施非常有帮助。衷心感谢您的合作!本次调查采用匿名方式，所有信息均严格保密，请放心填写。**

**第一部分:基本情况**

1. 年龄：_____

2. 性别：

①女

②男

3. 所在班级

①高中

②初中

4. 性格

①内向

②外向

5. 上学方式:

①走读

②住校

6. 目前居住在:

①城区

②乡镇

③农村

7. 家庭经济状况:

①好

②中

③差

8. 父母是否外出打工:

①都在外打工

②父亲或母亲外出打工

③都在家

9. 自认为的学习成绩:

①非常好

②中等

③差

10. 认为自己的学习压力:

①轻

②中等

③重

11. 学校是否组织过心理健康教育活动？

①经常

②偶尔

③从不

**第二部分: 中学生心理健康量表**

**这个测试是调查你的心情和感受的，不是测试智力和学习能力，与学习成绩无关，答案也没有好坏之分。**

**①请按照你平常所想的如实回答；**

**②每一问题都要回答，但只能选择一个答案；难以决定时，请选与你最接近的答案；**

**③不明白的地方可以举手问老师；**

**④回答时间没有限制，但不要过分考虑，请写出你最初想要的答案；**

**⑤本测试每一问题都只有“是”和“否”两种可供选择的答案。**

**请把你所选的答案在相应的位置上画上√。**

| **中学生心理健康量表** | **是** | **不是** |
| --- | --- | --- |
| 1. 你晚上要睡觉时，是否总想着明天的功课? |  |  |
| 1. 老师向全班提问时，你是否会觉得是在问自己而感到不安? |  |  |
| 1. 你是否一听说“要考试”心里就紧张。 |  |  |
| 1. 你考试成绩不好时，心里是否感到很不快? |  |  |
| 1. 你学习成绩不好时，是否总是提心吊胆? |  |  |
| 1. 你考试时，想不起原来掌握的知识时，是否会感到紧张不安？ |  |  |
| 1. 你考试后，在没有知道成绩之前，是否总是放心不下? |  |  |
| 1. 你是否一遇到考试，就担心会考坏? |  |  |
| 1. 你是否希望每次考试都能顺利? |  |  |
| 1. 你在没有完成任务之前，是否总担心完不成任务？ |  |  |
| 1. 你当着大家面朗读课文时，是否总是怕读错? |  |  |
| 1. 你是否认为学校里得到的学习成绩总是不大可靠? |  |  |
| 1. 你是否认为你比别人更担心学习? |  |  |
| 1. 你是否做过考试考坏了的梦? |  |  |
| 1. 你是否做过学习成绩不好时，受到爸爸妈妈或老师训斥的梦? |  |  |
| 1. 你是否经常觉得有同学在背后说你的坏话? |  |  |
| 1. 你受到父母批评后，是否总是想不开，放在心上? |  |  |
| 1. 你在游戏或与别人的竞争中输给了对方，是否就不想再干了? |  |  |
| 1. 人家在背后议论你，你是否感到讨厌? |  |  |
| 1. 你在大家面前或被老师提问时，是否会脸红? |  |  |
| 1. 你是否很担心叫你担任班级工作? |  |  |
| 1. 你是否总是觉得好像有人在注意你? |  |  |
| 1. 你在工作或学习时，如果有人在注意你，你心里是否会紧张? |  |  |
| 1. 你受到批评时，心情是否不愉快? |  |  |
| 1. 你受到老师批评时，心里是否总是不安? |  |  |
| 1. 同学们在笑时，你是否也不大会笑? |  |  |
| 1. 你是否觉得到同学家里去玩时不如在自己家里玩? |  |  |
| 1. 你和大家在一起时，是否也觉得自己是孤单的一个人? |  |  |
| 1. 你是否觉得和同学一起玩，不如自己—个人玩? |  |  |
| 1. 同学们在交谈时，你是否不想加入? |  |  |
| 1. 你和大家在—起时，是否觉得自己是多余的人? |  |  |
| 1. 你是否讨厌参加运动会和文艺演出? |  |  |
| 1. 你的朋友是否很少? |  |  |
| 1. 你是否不喜欢同别人谈话? |  |  |
| 1. 在人多的地方，你是否觉得很怕? |  |  |
| 1. 你在参加排球、篮球等集体比赛输了时，心里是否一直认为自己没做好? |  |  |
| 1. 你受到批评后，是否总认为是自己不好? |  |  |
| 1. 别人笑你的时候，你是否会认为是自己做错了什么事? |  |  |
| 1. 你学习成绩不好时，是否总是认为是自己不用功的缘故? |  |  |
| 1. 你做事失败的时候，是否总是认为是自己的责任? |  |  |
| 1. 大家受到责备时，你是否认为主要是自己的过错? |  |  |
| 1. 你参加乒乓球、羽毛球、广播操等体育比赛时，是否一出错就特别留神? |  |  |
| 1. 碰到为难的事情时，你是否认为自己难以应付? |  |  |
| 1. 你是否有时会后悔：“那件事不做就好了”? |  |  |
| 1. 你和同学吵架以后，是否总是认为是自己的错? |  |  |
| 1. 你心里是否总想为班级做点好事? |  |  |
| 1. 你学习的时候，思想是否经常开小差? |  |  |
| 1. 你把东西借给别人时，是否担心别人会把东西弄坏? |  |  |
| 1. 碰到不顺利的事情时，你心里是否很烦躁? |  |  |
| 1. 你是否非常担心家里有人生病或死去? |  |  |
| 1. 你是否在梦里见到过死去的人? |  |  |
| 1. 你对收音机和汽车的声音是否特别敏感? |  |  |
| 1. 你心里是否总觉得好像有什么事没有做好? |  |  |
| 1. 你是否总担心会发生什么意外的事? |  |  |
| 1. 你在决定要做什么事时，是否总是犹豫不决? |  |  |
| 1. 你手上是否经常出汗? |  |  |
| 1. 你害羞时是否会脸红? |  |  |
| 1. 你是否经常头痛? |  |  |
| 1. 你被老师提问时，心里是否总是很紧张? |  |  |
| 1. 你没有参加运动，心脏是否经常扑腾扑腾地跳? |  |  |
| 1. 你是否很容易疲劳? |  |  |
| 1. 你是否很不愿吃药? |  |  |
| 1. 夜里你是否很难入睡? |  |  |
| 1. 你是否总觉得身体好像有什么毛病? |  |  |
| 1. 你是否经常认为自己的体型和面孔比别人难看? |  |  |
| 1. 你是否经常觉得肠胃不好? |  |  |
| 1. 你是否经常咬指甲? |  |  |
| 1. 你是否经常舔手指头? |  |  |
| 1. 你是否经常感到呼吸困难? |  |  |
| 1. 你去厕所的次数是否比别人多? |  |  |
| 1. 你是否很怕到高的地方去? |  |  |
| 1. 你是否害怕很多东西? |  |  |
| 1. 你是否经常做噩梦? |  |  |
| 1. 你胆子是否很小? |  |  |
| 1. 夜里，你是否很怕一个人在房间里睡觉? |  |  |
| 1. 你乘车穿过隧道或路过高桥时，是否很怕? |  |  |
| 1. 你是否喜欢整夜开着灯睡觉? |  |  |
| 1. 你听到打雷声是否非常害怕? |  |  |
| 1. 你是否非常害怕黑暗? |  |  |
| 1. 你是否经常感到后面有人跟着你? |  |  |
| 1. 你是否经常生气? |  |  |
|  |  |  |
| 1. 你是否不想得到好的成绩? |  |  |
| 1. 你是否经常会突然想哭? |  |  |
| 1. 你以前是否说过谎话? |  |  |
| 1. 你有时是否会觉得，还是死了好? |  |  |
| 1. 你是否一次也没有失约过? |  |  |
| 1. 你是否经常想大声喊叫? |  |  |
| 1. 你是否能保密别人不让说的事? |  |  |
| 1. 你有时是否想过自己一个人到远的地方去? |  |  |
| 1. 你是否总是很有礼貌? |  |  |
| 1. 你被人说了坏话，是否想立即采取报复行动? |  |  |
| 1. 老师或父母说的话，你是否都照办? |  |  |
| 1. 你心里不开心，是否会乱丢、乱砸东西? |  |  |
| 1. 你是否发过怒? |  |  |
| 1. 你想要的东西，是否就—定要拿到手? |  |  |
| 1. 你不喜欢的功课老师提前下课，你是否会感到特别高兴? |  |  |
| 1. 你是否经常想从高的地方跳下来? |  |  |
| 1. 你是否无论对谁都很亲热? |  |  |
| 1. 你是否会经常急躁得坐立不安? |  |  |
| 100.对不认识的人，你是否会都喜欢? |  |  |
