# Supplementary material for: Use of latent profile analysis and k-means clustering to identify student anxiety profiles
Source: BMC Psychiatry. 2022 Jan 5;22:12. doi: 10.1186/s12888-021-03648-7 (PMC8728926; doi:10.1186/s12888-021-03648-7)
Supplement: Supplementary file 2 — Additional file 2. Univariate analysis of different latent profiles of anxiety students [file 12888_2021_3648_MOESM2_ESM.docx]

| Variable | The low-risk group (N=2874) | The average-risk group (N=3713) | The high-risk group (N=3151) | χ^2^ | P |
| --- | --- | --- | --- | --- | --- |
| ***Gender*** |  |  |  |  |  |
| Female | 1155 | 1846 | 1972 | 306.067 | <0.001 |
| Male | 1719 | 1867 | 1179 |  |  |
| ***Grade*** |  |  |  |  |  |
| Senior | 923 | 805 | 740 | 101.744 | <0.001 |
| Junior | 1951 | 2908 | 2411 |  |  |
| **Character traits** |  |  |  |  |  |
| Introversion | 916 | 1733 | 1370 | 155.801 | <0.001 |
| Extroversion | 1958 | 1980 | 1781 |  |  |
| ***Mode of travel to school*** |  |  |  |  |  |
| Nonresident | 1952 | 2580 | 2035 | 19.091 | <0.001 |
| In-residence | 922 | 1133 | 1116 |  |  |
| ***Residence*** |  |  |  |  |  |
| Urban area | 1383 | 1423 | 1183 | 96.340 | <0.001 |
| Town | 443 | 634 | 628 |  |  |
| Rural area | 1048 | 1656 | 1340 |  |  |
| ***Family financial conditions*** | |  |  |  |  |
| Good | 140 | 136 | 91 | 50.251 | <0.001 |
| Average | 2228 | 2755 | 2304 |  |  |
| Poor | 506 | 822 | 756 |  |  |
| ***Whether parents work outside*** | |  |  |  |  |
| Both outside | 1114 | 1578 | 1338 | 27.441 | <0.001 |
| Father or mother outside | 779 | 1056 | 912 |  |  |
| Both at home | 981 | 1079 | 901 |  |  |
| ***Academic performance*** |  |  |  |  |  |
| Upper | 487 | 396 | 262 | 298.051 | <0.001 |
| Medium | 1626 | 1963 | 1434 |  |  |
| Lower | 761 | 1354 | 1455 |  |  |
| ***Perceived academic pressure*** | |  |  |  |  |
| Light | 539 | 325 | 246 | 550.498 | <0.001 |
| Average | 1779 | 2113 | 1492 |  |  |
| Heavy | 556 | 1275 | 1413 |  |  |
| ***Has the school organized mental health education activities*** | | | | | |
| Often | 1318 | 1253 | 852 | 291.053 | <0.001 |
| Occasionally | 1286 | 2007 | 1714 |  |  |
| Never | 270 | 453 | 585 |  |  |

**Additional File 2** Univariate analysis of different latent profiles of anxiety students
